# Associations of eHealth Literacy with Diabetes Self-Efficacy and Glycemic Parameters in Individuals with Type 2 Diabetes and Prediabetes: A Cross-Sectional Study

**DOI:** 10.3390/healthcare14172806

**Published:** 2026-09-01

**Authors:** Kemal Ozan Lüle, Sümeyye Akçoban, Aylin Berk, Neşe Ataman Bor, Soner Berşe

**Affiliations:** 1 Department of Internal Medicine, Faculty of Medicine, Gaziantep University, Gaziantep 27310, Türkiye; 2 Kırıkhan Vocational School of Health Services, Hatay Mustafa Kemal University, Hatay 31440, Türkiye; sumeyyea2016@gmail.com; 3 Nursing Graduate Program, Institute of Health Sciences, Gaziantep University, Gaziantep 27310, Türkiye; 4 Department of Nursing, Faculty of Health Sciences, Hakkari University, Hakkari 30000, Türkiye; 5 Department of Nursing, Faculty of Health Sciences, Gaziantep University, Gaziantep 27310, Türkiye

**Keywords:** eHealth literacy, type 2 diabetes mellitus, prediabetes, self-efficacy, glycemic control

## Abstract

**Highlights:**

**What are the main findings?**

- eHealth literacy was positively associated with diabetes self-efficacy.
- Higher self-efficacy was associated with lower HbA1c among participants with type 2 diabetes.
- Longer disease duration was associated with higher HbA1c.
- A statistical indirect association between eHealth literacy and HbA1c through self-efficacy was observed.

**What are the implications of the main findings?**

- Digital health literacy assessment may help inform diabetes education strategies.
- Diabetes education may benefit from integrating digital health literacy support with strategies aimed at strengthening self-efficacy.

**Abstract:**

Background/Objectives: eHealth literacy may support diabetes self-management, but its relationships with diabetes self-efficacy and glycemic outcomes remain unclear. This study examined these associations among adults with type 2 diabetes mellitus (T2DM) and prediabetes and evaluated whether the observed association between eHealth literacy and HbA1c was statistically consistent with an indirect association through diabetes self-efficacy. Methods: This cross-sectional study included 273 adults with T2DM or prediabetes attending an internal medicine outpatient clinic in Türkiye. Data were collected using the eHealth Literacy Scale, Diabetes Self-Efficacy Scale, and a clinical characteristics form; HbA1c and fasting plasma glucose values were obtained from medical records. Correlation, regression, and bootstrapped statistical indirect-association analyses were performed. Results: eHealth literacy was positively associated with diabetes self-efficacy (rho = 0.310, *p* < 0.001; β = 0.345, *p* < 0.001). Its association with HbA1c was non-significant after adjustment. Among participants with T2DM, higher self-efficacy was associated with lower HbA1c (β = −0.224, *p* = 0.026), whereas longer disease duration was associated with higher HbA1c (β = 0.201, *p* = 0.023). A significant statistical indirect association between eHealth literacy and HbA1c through diabetes self-efficacy was observed. Conclusions: This study provides correlational evidence that eHealth literacy is associated with diabetes self-efficacy and that this relationship may partly account for the observed statistical association between eHealth literacy and glycemic control. The cross-sectional design precludes conclusions regarding temporal or causal mediation.

## 1. Introduction

Type 2 diabetes and prediabetes are increasingly prevalent metabolic conditions characterized by impaired glucose regulation and substantial long-term health consequences. Prediabetes, defined by glucose levels above normal but below diagnostic thresholds for diabetes, represents a high-risk state for progression to diabetes [1,2,3]. Once diabetes develops, sustained hyperglycemia may lead to serious complications, including cardiovascular disease, neuropathy, nephropathy, and retinopathy. Therefore, preventing disease progression depends not only on clinical interventions but also on individuals’ capacity to understand, interpret, and apply health-related information. The literature consistently demonstrates that health literacy is a key determinant of risk perception, self-management behaviors, and the adoption of preventive health practices [4,5,6].

Health literacy refers to the ability to access, comprehend, evaluate, and use health information in decision-making processes, and low levels have been associated with adverse outcomes in the management of chronic diseases [5]. In diabetes and prediabetes, health literacy is critical for risk awareness, treatment adherence, and the maintenance of lifestyle modifications, and health literacy-based interventions have been shown to improve self-management behaviors and glycemic control [6,7]. Moreover, higher health literacy has been reported to be associated with better glycemic outcomes [6,8].

With the widespread adoption of digital health technologies, the digital dimension of health literacy, eHealth literacy (eHL), has gained increasing importance. eHL encompasses individuals’ abilities to seek, appraise, and apply health information obtained from online sources [9]. Higher levels of eHL have been associated with better self-care behaviors and improved health outcomes in individuals with diabetes [10]. In addition, emerging evidence suggests that eHL may be indirectly associated with self-management behaviors through psychosocial factors such as self-efficacy [11,12].

The present study is grounded in Social Cognitive Theory (SCT), which posits reciprocal relationships among personal cognitive factors, behavioral patterns, and environmental influences [13]. Within this theoretical framework, eHealth literacy can be conceptualized as an environmental and cognitive resource related to access to, understanding of, and use of health information. For individuals with type 2 diabetes or prediabetes, perceived ability to locate and evaluate digital health information may be associated with confidence in disease management and self-management behaviors [10,11,14].

Self-efficacy is a central SCT construct and refers to an individual’s belief in their capacity to perform behaviors necessary to achieve desired outcomes [13]. In diabetes management, self-efficacy is theorized to be related to lifestyle adherence, glucose monitoring, treatment-related decisions, and other self-management behaviors [14,15]. These theoretical relationships provide the conceptual basis for examining whether the association between eHealth literacy and HbA1c is statistically consistent with an indirect association through diabetes self-efficacy [11,14,15].

A strong association between health literacy and self-efficacy has been reported in the literature, and higher self-efficacy is consistently linked to more effective diabetes self-management behaviors and better glycemic control [15]. In this context, eHealth literacy may be conceptually related to glycemic parameters primarily through its close relationship with individuals’ confidence and capability to manage their condition, rather than through a direct clinical effect [8,10].

Despite growing interest, studies simultaneously examining eHL, biopsychosocial determinants, and clinical outcomes remain limited [14,16]. Bibliometric analyses further indicate that research on health literacy in the context of diabetes is still developing [17]. In Turkey, eHL research is limited, and existing studies predominantly focus on populations with diabetes [18,19,20]. While the global literature largely concentrates on established diabetes, prediabetes represents a critical window for intervention before disease progression. Examining both conditions within a single study allows for comparison of eHealth literacy patterns across the glycemic continuum, from at-risk (prediabetes) to established disease (diabetes), and may inform stage-specific intervention strategies. This approach is supported by recent calls in the literature for integrated prevention-management frameworks that bridge the prediabetes–diabetes divide [2,3]. Early-onset type 2 diabetes is increasingly recognized, and age at diagnosis has been associated with heterogeneous vascular and microvascular outcomes [21,22].

Based on Social Cognitive Theory and the existing literature, the present study aimed to (1) assess eHealth literacy and diabetes self-efficacy among individuals with prediabetes and type 2 diabetes; (2) compare eHealth literacy, diabetes self-efficacy, and glycemic characteristics between the groups; (3) examine associations among eHealth literacy, diabetes self-efficacy, and glycemic parameters; (4) identify variables associated with diabetes self-efficacy and HbA1c; and (5) evaluate whether the relationship between eHealth literacy and HbA1c was statistically consistent with an indirect association through diabetes self-efficacy.

## 2. Methods

### 2.1. Study Design and Setting

This descriptive cross-sectional study was conducted at the Department of Internal Medicine of Gaziantep University Şahinbey Research and Practice Hospital, Gaziantep, Türkiye. The study examined the associations between eHealth literacy and diabetes self-efficacy, as well as their relationships with glycemic parameters, including glycated hemoglobin (HbA1c) and fasting plasma glucose, among adults with type 2 diabetes and prediabetes. The study was reported in accordance with the Strengthening the Reporting of Observational Studies in Epidemiology (STROBE) guidelines for cross-sectional studies.

### 2.2. Ethical Considerations

The study protocol was approved by the Hakkari University Scientific Research and Publication Ethics Committee (Decision No: 2025/275-5; Date: 26 December 2025). Institutional permission was obtained from the Gaziantep University Şahinbey Research and Practice Hospital Administration. All participants provided written informed consent before enrollment. The study was conducted in accordance with the principles of the Declaration of Helsinki. All study hypotheses, variables, and analytical procedures were specified a priori in the research protocol submitted for ethics review before the initiation of data collection.

### 2.3. Participants

The study population consisted of adults aged 18 years or older with type 2 diabetes or prediabetes attending the Department of Internal Medicine outpatient clinic. Glycemic status was classified according to the American Diabetes Association (ADA) 2024 criteria [23]. Type 2 diabetes was defined as HbA1c 6.5% or higher or fasting plasma glucose (FPG) 126 mg/dL or higher; prediabetes was defined as HbA1c 5.7–6.4% or FPG 100–125 mg/dL. In cases of discordance between HbA1c and FPG values, the higher-risk category was assigned.

Exclusion criteria included age younger than 18 years, normal glycemic status (HbA1c less than 5.7% and FPG less than 100 mg/dL), type 1 diabetes, gestational diabetes, cognitive impairment precluding informed consent, inability to complete the questionnaires, and incomplete glycemic data. Internet use or previous use of digital health information was not used as an exclusion criterion. This decision was made because the study aimed to assess eHealth literacy in a real-world outpatient population, including individuals with varying levels of digital exposure. Excluding individuals with limited or no internet use could have artificially restricted the sample to digitally active participants and reduced the representativeness of the clinical population.

A convenience sampling method was employed. Of 282 patients initially screened, 5 participants aged less than 18 years, 1 participant with normal glycemic status, and 3 participants with incomplete glycemic data were excluded, yielding a final analytic sample of *N* = 273 adults (type 2 diabetes: *n* = 163, 59.7%; prediabetes: *n* = 110, 40.3%).

No a priori sample size calculation was performed before data collection. The sample size was determined by the number of eligible and consenting patients who attended the outpatient clinic during the planned data collection period from January to March 2026. This approach was used because the study was designed as a single-center cross-sectional study using convenience sampling. To evaluate the adequacy of the achieved sample size, we conducted a post hoc power analysis using G*Power version 3.1.9.7 principles. For the regression model with the largest number of predictors (Model 2: k = 7, *n* = 134), the observed R^2^ = 0.155 yielded a Cohen’s f^2^ = 0.183, corresponding to a medium effect, with statistical power exceeding 0.99 at α = 0.05. For Model 1 (k = 4, *N* = 273, R^2^ = 0.102), power also exceeded 0.99. These results indicate that the achieved sample size was adequate for detecting the associations of the observed magnitudes; however, because the analysis was post hoc, it should be interpreted as a sensitivity assessment rather than as prospective sample size justification. Statistical power does not establish clinical importance or causal interpretation.

### 2.4. Data Collection

Data were collected through face-to-face interviews conducted by researchers between January and March 2026 at the outpatient clinic. Each interview lasted approximately 15–20 min. The data collection form included sociodemographic characteristics (age, gender, education level), clinical parameters obtained from medical records (HbA1c, FPG), anthropometric measurements (BMI, waist circumference), disease duration (years since diagnosis), presence of comorbidities, and two validated self-report scales (eHEALS and DSES). Because the eHEALS evaluates perceived ability to find, evaluate, and use online health information, all participants were asked to complete the scale regardless of their level of internet use. The two non-scored internet-use items of the eHEALS were retained to capture participants’ general exposure to online health information. Participants were not analyzed separately according to internet-use status because the main objective was to evaluate eHealth literacy as a continuous construct across the full clinical sample. This issue was also acknowledged in the Limitations section. HbA1c values were extracted from laboratory results documented in the clinical records and were not self-reported. The exact interval between the HbA1c measurement and questionnaire administration was not recorded as a separate variable.

### 2.5. Instruments

#### 2.5.1. Ehealth Literacy Scale (Eheals)

The eHealth Literacy Scale (eHEALS) was used to assess participants’ perceived skills in using information technology for health. The scale was originally developed by Norman and Skinner [24], who reported a Cronbach’s alpha coefficient of 0.88. The Turkish validity and reliability study was conducted by Uskun et al. [25], reporting a Cronbach’s alpha of 0.97. The scale consists of 10 items in total. Two items evaluate internet use (non-scored), and eight items measure e-health literacy. Responses are rated on a 5-point Likert scale ranging from 1 (Strongly Disagree) to 5 (Strongly Agree). The total score ranges from 8 to 40, with higher scores indicating higher eHealth literacy. It should be noted that the eHEALS measures perceived, rather than actual, eHealth skills [9,26]. The implications of this distinction are addressed in the limitations section. In the present study, the scale demonstrated excellent internal consistency (Cronbach’s alpha = 0.951).

#### 2.5.2. Diabetes Self-Efficacy Scale (Dses)

Diabetes self-efficacy was assessed using the Turkish version of the Diabetes Self-Efficacy Scale (DSES), adapted by Mankan et al. [27]. The scale consists of 8 items rated on a 10-point scale ranging from 1 (not at all confident) to 10 (totally confident). The total score ranges from 8 to 80, with higher scores indicating greater self-efficacy. Mankan et al. (2017) [27] reported a Cronbach’s alpha of 0.86. In the present study, the scale demonstrated good internal consistency (Cronbach’s alpha = 0.865).

### 2.6. Clinical Parameters

Glycated hemoglobin (HbA1c, %) and fasting plasma glucose (FPG, mg/dL) values were obtained from laboratory results documented in participants’ clinical records and were not self-reported. The exact interval between HbA1c measurement and administration of eHEALS and DSES was not recorded as a separate variable. Body mass index (BMI, kg/m^2^) was calculated from measured height and weight. Waist circumference (cm) was measured at the midpoint between the lowest rib and the iliac crest and was included for descriptive characterization; it was not entered as an explanatory variable in the regression models.

### 2.7. Statistical Analysis

Data were analyzed using IBM SPSS Statistics version 26.0 (IBM Corp., Armonk, NY, USA) [28]. Descriptive statistics were presented as mean, standard deviation, minimum, maximum, skewness, and kurtosis for continuous variables and as frequencies and percentages for categorical variables. Normality was assessed using the Shapiro–Wilk test, histograms, Q–Q plots, and skewness and kurtosis values. Extreme values were examined using boxplots and standardized values before selecting the statistical tests. Values were checked against the original data and retained in the analyses when they represented plausible clinical observations rather than data-entry errors. Because several key variables continued to deviate from normal distribution after this assessment, non-parametric tests were used for bivariate group comparisons and correlation analyses. Multicollinearity was assessed using VIF values. Formal diagnostic results for linearity, homoscedasticity, residual normality, and influential observations were not retained in the analysis output available for this revision; regression findings should therefore be interpreted with this limitation in mind.

Internal consistency was evaluated using Cronbach’s alpha and item-total correlations. Comparisons between diabetes and prediabetes groups were performed using the Mann–Whitney U test for continuous variables and the chi-square test for categorical variables. Rank-biserial correlation (|r|) was reported as the effect size for Mann–Whitney U tests, and Cramér’s V was reported as the effect size for chi-square tests. Because HbA1c and FPG were used to define glycemic status, inferential tests for these variables were not considered meaningful (tautological) and were therefore not reported.

Spearman rank correlations (rho) were computed with 95% bootstrap confidence intervals (1000 resamples). The correlation analysis included age, BMI, waist circumference, disease duration, eHealth literacy, diabetes self-efficacy, HbA1c, and FPG in order to provide a more comprehensive overview of the bivariate associations among continuous study variables. Partial correlations controlling for age and education were calculated to address potential confounding. Age and education were selected as control variables based on their established associations with both eHealth literacy and glycemic outcomes in the literature [10,29], and their significant bivariate correlations with key study variables in preliminary analyses. Pairwise deletion was used for correlation analyses, and the sample size for each correlation was reported because the number of available observations differed across variables.

Multiple linear regression analyses examined variables associated with diabetes self-efficacy and HbA1c. Model 1 used the full sample (*N* = 273) and included age, gender, education level, and eHealth literacy. Model 2 was restricted to participants with type 2 diabetes because HbA1c was treated as a clinical outcome in established diabetes; it included age, gender, education, eHealth literacy, DSES, disease duration, and comorbidity status.

Model 2 used a diabetes-only complete-case sample. Of 163 participants with type 2 diabetes, 29 were excluded by listwise deletion because HbA1c and/or disease-duration data were missing, resulting in *n* = 134. Age, gender, education, eHEALS, DSES, and comorbidity status were complete. The available aggregate data did not permit the 29 exclusions to be separated into mutually exclusive variable-specific categories because some participants could have missing values for both HbA1c and disease duration.

VIF values were examined for multicollinearity (criterion: <10; all observed VIF values < 2.8). Model 1 included *N* = 273 and Model 2 included *n* = 134 complete cases. Multiple imputation was not performed because the principal self-report variables and all Model 1 variables were complete, while missingness was concentrated in selected clinical variables. A formal MCAR assessment was not conducted.

A bootstrapped statistical indirect-association analysis (5000 resamples) was conducted exclusively in the type 2 diabetes subgroup using complete cases for the included variables (*n* = 134), controlling for age and education. Bootstrapping improves estimation of the statistical indirect association and its uncertainty but does not establish temporal precedence or causal mediation. Statistical significance was set at *p* < 0.05 (two-tailed).

## 3. Results

### 3.1. Sample Characteristics

The final sample consisted of 273 adults with type 2 diabetes (*n* = 163, 59.7%) or prediabetes (*n* = 110, 40.3%). As shown in Table 1, mean age was 38.28 ± 14.79 years, with 62.3% of participants being male. Nearly half of the participants had university or graduate education. Mean HbA1c was 7.40 ± 1.86%, and mean fasting plasma glucose was 144.91 ± 57.18 mg/dL. Mean eHEALS score was 25.77 ± 7.56, indicating moderate perceived eHealth literacy, whereas mean DSES score was 59.66 ± 12.93, indicating moderate-to-high diabetes self-efficacy. Mean disease duration was 5.86 ± 6.72 years, and comorbidities were present in 40.3% of participants.

Missing observations in the total sample were: HbA1c, 11/273 (4.0%); FPG, 7/273 (2.6%); BMI, 5/273 (1.8%); waist circumference, 29/273 (10.6%); and disease duration, 21/273 (7.7%). Age, gender, education, eHEALS, DSES, and comorbidity status were complete. Model 1 included all 273 participants. Model 2 and the statistical indirect-association analysis were restricted to participants with type 2 diabetes and used 134 complete cases.

### 3.2. Scale Reliability

Both scales demonstrated good to excellent internal consistency. The eHEALS showed excellent reliability (α = 0.951) with item-total correlations ranging from 0.786 to 0.879. The DSES demonstrated good reliability (α = 0.865) with item-total correlations ranging from 0.468 to 0.645.

### 3.3. Comparison: Diabetes vs. Prediabetes

Formal group comparisons were conducted between participants with prediabetes (*n* = 110) and type 2 diabetes (*n* = 163). As shown in Table 2, the prediabetes group had lower HbA1c (6.02 ± 0.24% vs. 8.38 ± 1.89%) and FPG (108.50 ± 8.40 vs. 170.21 ± 62.71 mg/dL), as expected from the classification criteria; statistical tests are not reported for these variables.

The prediabetes group showed significantly higher DSES scores (63.64 ± 11.66 vs. 56.97 ± 13.08, U = 11,735.5, *p* < 0.001, |r| = 0.31), a medium effect indicating greater self-efficacy. The prediabetes group was also significantly older (41.75 ± 14.09 vs. 35.94 ± 14.84, *p* = 0.001, |r| = 0.23), had higher BMI (28.76 ± 6.28 vs. 25.92 ± 6.14, *p* < 0.001, |r| = 0.28), higher waist circumference (94.96 ± 17.64 vs. 92.01 ± 30.46, *p* = 0.026, |r| = 0.17), and shorter disease duration (2.64 ± 4.01 vs. 8.28 ± 7.32, *p* < 0.001, |r| = 0.53). No significant differences were found for eHEALS (*p* = 0.111) or gender (χ^2^ = 0.58, *p* = 0.445, V = 0.05).

### 3.4. Correlation Analysis

The correlation analysis included age, eHealth literacy, diabetes self-efficacy, HbA1c, and FPG. These variables were selected because they corresponded directly to the principal study relationships involving eHealth literacy, self-efficacy, and glycemic outcomes. Anthropometric variables and disease duration were examined descriptively and in the relevant group or multivariable analyses but were not included in this focused correlation matrix.

Spearman correlations revealed significant associations (Table 3). eHealth literacy showed a moderate positive correlation with DSES (rho = 0.310, *p* < 0.001). Age was moderately negatively correlated with eHealth literacy (rho = −0.350, *p* < 0.001).

A weak positive correlation between eHealth literacy and HbA1c (rho = 0.176, *p* = 0.004) became non-significant after controlling for age and education (partial r = 0.035, *p* = 0.574), suggesting that age may account for part of the unadjusted association. After adjustment, DSES was negatively correlated with HbA1c (r = −0.211, *p* < 0.001) and FPG (r = −0.234, *p* < 0.001). These statistical associations should not be interpreted as evidence of causality or clinically meaningful improvement.

### 3.5. Multiple Regression Analysis

Two regression analyses addressed distinct outcomes and populations. Model 1 examined variables associated with diabetes self-efficacy in the total sample (*N* = 273). Model 2 examined variables associated with HbA1c exclusively among participants with type 2 diabetes who had complete data for all included variables (*n* = 134).

As shown in Table 4, Model 1 (DSES, *N* = 273) was significant (F(4, 268) = 7.58, *p* < 0.001, R2 = 0.102). eHealth literacy was the only variable significantly associated with DSES (B = 0.589, β = 0.345, *p* < 0.001); each 1-point increase in eHEALS was associated with a 0.59-point higher DSES. All VIF values were below 1.6, indicating no multicollinearity.

For the diabetes-specific HbA1c analysis, the basic model included the principal study variables and sociodemographic covariates (age, gender, education, eHealth literacy, and self-efficacy). The expanded model additionally included disease duration and comorbidity because these variables are specific clinical characteristics of participants with type 2 diabetes and may be associated with glycemic control.

As shown in Table 5, the expanded Model 2 (HbA1c, diabetes-only *n* = 134) was significant (F(7, 126) = 3.29, *p* = 0.003, R^2^ = 0.155) and explained more variance than the basic model without disease duration and comorbidity (R^2^ = 0.103).Thus, the expanded model represents adjustment for additional diabetes-related clinical factors rather than the addition of variables solely to increase explained variance. DSES was significantly negatively associated with HbA1c (B = −0.031, β = −0.224, *p* = 0.026), and disease duration was significantly positively associated with HbA1c (B = 0.053, β = 0.201, *p* = 0.023). Age was no longer significant when duration was included (*p* = 0.232). eHealth literacy was not directly associated with HbA1c (*p* = 0.474). All VIF values were below 2.8.

### 3.6. Statistical Indirect-Association Analysis

The analysis was conducted among 134 participants with type 2 diabetes with complete data for the included variables. A bootstrapped statistical indirect-association analysis with 5000 resamples controlled for age and education (Table 6). eHEALS was associated with DSES (a = 0.644, SE = 0.115, *p* < 0.001), and DSES was associated with HbA1c after accounting for eHEALS and the covariates (b = −0.034, SE = 0.009, *p* < 0.001). The direct association between eHEALS and HbA1c (c′ = 0.024) was not significant. The estimated statistical indirect association was −0.022 (95% bootstrap CI: −0.039 to −0.009; Sobel z = −3.167, *p* = 0.002). These findings are statistically consistent with an indirect association through self-efficacy; they do not establish temporal precedence or causal mediation.

In summary, eHealth literacy was positively associated with diabetes self-efficacy in the total sample. In the diabetes-only regression, self-efficacy and disease duration were significantly associated with HbA1c. The bootstrapped analysis identified a statistical indirect association between eHealth literacy and HbA1c through self-efficacy. The cross-sectional design precludes temporal or causal interpretation.

## 4. Discussion

This study was conducted to evaluate the associations between eHealth literacy, diabetes self-efficacy, and glycemic parameters in individuals with diabetes and prediabetes, guided by Social Cognitive Theory (SCT). The findings are discussed in light of the relevant literature. Model 1 concerned the full sample, whereas Model 2 and the statistical indirect-association analysis were restricted to participants with type 2 diabetes.

In this study, perceived eHealth literacy was moderate (eHEALS mean = 25.77 ± 7.56), consistent with previous diabetes studies [10,30,31]. However, eHEALS measures perceived ability rather than objectively demonstrated digital health performance [9,24,26]. The score should therefore not be interpreted as evidence of competence or deficiency in specific skills such as critical appraisal or practical application. Performance-based and multidimensional measures are required to evaluate those domains directly.

The mean DSES score in our study (59.66 ± 12.93) was found to be at a moderate-to-high level. Consistent with this finding, Ataya et al. reported moderate-to-high self-efficacy levels using the Diabetes Management Self-Efficacy Scale in individuals with diabetes [32]. Similarly, Eshtaya et al. found that participants’ self-efficacy scores were above the moderate level in their study evaluating diabetes management self-efficacy [33]. In line with these literature findings, the moderate-to-high level of diabetes self-efficacy identified in our study indicates that individuals with diabetes and prediabetes generally have positive self-efficacy perceptions regarding disease management.

A moderate positive association was found between eHealth literacy and diabetes self-efficacy (rho = 0.310, *p* < 0.001). This finding is consistent with studies demonstrating positive correlations between eHealth literacy and self-efficacy [11,30]. In our initial correlation analysis, a weak positive association between eHealth literacy and HbA1c was observed. However, this association became non-significant when confounding variables such as age and education were controlled. Stephen et al. reported that the associations observed between eHealth literacy and HbA1c in adults with type 1 diabetes were attenuated when age and other demographic characteristics were controlled for in the model [29]. Similarly, AlShehri and Khojah reported that the association between eHealth literacy and self-care activities in individuals with type 2 diabetes was influenced by demographic characteristics [10]. These results indicate that eHealth literacy is more closely associated with cognitive processes such as diabetes self-efficacy than with glycemic control directly. Demographic characteristics such as age and education should be taken into account when evaluating glycemic outcomes.

The positive association between eHealth literacy and diabetes self-efficacy can be interpreted within the framework of SCT. Accessing and understanding reliable digital health information may increase individuals’ knowledge of diabetes, improve their awareness of available self-management strategies, and strengthen their confidence in performing disease-management activities. Within this framework, eHealth literacy may function as a cognitive and informational resource that supports self-efficacy rather than exerting a direct biological effect on glycemic control. This interpretation is consistent with previous studies showing that eHealth literacy is associated with diabetes self-efficacy, self-care behaviors, and self-management [11,12,14].

The absence of a significant direct association between eHealth literacy and HbA1c after adjustment does not necessarily indicate that eHealth literacy is clinically unimportant. Digital health information is unlikely to influence glycemic outcomes unless that information is understood, considered trustworthy, translated into appropriate decisions, and incorporated into sustained self-management behaviors. Self-efficacy may therefore represent one of the mechanisms through which eHealth literacy is associated with glycemic outcomes. However, because the present study did not directly assess all relevant self-management behaviors, this explanation should be considered theoretically plausible rather than a demonstrated behavioral mechanism.

In the regression analyses, eHealth literacy was significantly associated with self-efficacy (β = 0.345, *p* < 0.001), a pattern compatible with SCT and previous studies [11,14]. The present cross-sectional findings identify eHealth literacy as a potentially modifiable correlate of self-efficacy, but they do not demonstrate that improving eHealth literacy will cause improvements in self-efficacy, self-management, or glycemic outcomes. Longitudinal and intervention studies are required to test that possibility.

From a clinical perspective, individuals with higher eHealth literacy may be better able to identify reliable digital resources, understand diabetes-related recommendations, communicate with healthcare professionals, and make informed self-management decisions. Nevertheless, access to information alone may be insufficient. Individuals must also feel confident in their ability to translate that information into daily behaviors. Therefore, diabetes education programs may be strengthened by combining digital health literacy support with strategies designed to increase self-efficacy.

The expanded Model 2 explained 15.5% of the variance in HbA1c, compared with 10.3% in the basic model. Longer disease duration was associated with higher HbA1c (β = 0.201, *p* = 0.023), a pattern that may be compatible with progressive beta-cell dysfunction [34]. After disease duration was included, age was no longer associated with HbA1c (*p* = 0.232), suggesting that disease duration may account for part of the apparent age-HbA1c relationship. Younger-onset diabetes may involve different disease characteristics or management challenges, but these possibilities cannot be distinguished by the present data. DSES was negatively associated with HbA1c (β = −0.224, *p* = 0.026); this does not establish a causal or clinically sufficient role for self-efficacy. Current age and disease duration were available, but age at diabetes diagnosis was not collected as a separate variable; therefore, early- versus later-onset diabetes could not be directly evaluated [21,22].

The negative association between self-efficacy and HbA1c suggests that confidence in performing diabetes-management activities may be clinically relevant to glycemic management. Individuals with greater self-efficacy may be more likely to follow dietary recommendations, engage in regular physical activity, monitor their glucose levels, take medications as prescribed, and maintain these behaviors when difficulties arise. Previous studies have similarly linked health literacy and self-efficacy with diabetes self-management and glycemic outcomes [7,15]. However, medication adherence, dietary behavior, physical activity, and glucose-monitoring practices were not directly measured in the present study. These behaviors should therefore be considered possible explanations for the observed association rather than mechanisms demonstrated by our findings.

The bootstrapped analysis identified a significant statistical indirect association (ab = −0.022, 95% bootstrap CI: −0.039 to −0.009): higher eHealth literacy was associated with greater self-efficacy, which was in turn associated with lower HbA1c. This pattern is compatible with SCT, prior correlational evidence [11,14], and estab-lished bootstrap indirect-effect methodology [35]. Nevertheless, bootstrapping quantifies the statistical association and uncertainty; it does not overcome the absence of temporal ordering or demonstrate causal mediation.

Theoretically, eHealth literacy may provide informational resources, whereas self-efficacy may help individuals translate information into self-management efforts. The observed statistical indirect association is compatible with this explanation. However, eHealth literacy and self-efficacy were measured concurrently, so temporal sequence and causal mediation cannot be established.

The comparison between diabetes and prediabetes groups revealed several notable differences. Individuals with prediabetes reported significantly higher self-efficacy levels (*p* < 0.001, |r| = 0.31), which may reflect the lower disease burden and fewer self-management demands in the prediabetes stage. The prediabetes group also had significantly shorter disease duration (2.64 vs. 8.28 years, *p* < 0.001), higher BMI (28.76 vs. 25.92, *p* < 0.001), and were older (41.75 vs. 35.94 years, *p* = 0.001). No significant differences were found in eHealth literacy levels, suggesting that perceived eHealth literacy levels may be comparable across the glycemic continuum. These findings highlight that self-efficacy interventions may be particularly important during the transition from prediabetes to diabetes, a period when self-management demands increase substantially.

The R^2^ values (10.2% for DSES and 15.5% for HbA1c) indicate modest explanatory power. Glycemic control is multifactorial, and eHealth literacy and self-efficacy represent only a limited component of factors associated with HbA1c. Medication adherence, treatment regimen, diet, physical activity, diabetes duration, socioeconomic circumstances, social support, psychological well-being, and healthcare access may contribute substantially. Statistical significance should not be interpreted as evidence that eHealth literacy or self-efficacy alone has sufficient explanatory, predictive, or clinical utility.

Taken together, the findings suggest that eHealth literacy may have clinical relevance primarily through its relationship with self-efficacy and subsequent self-management processes. In practice, merely directing patients to digital information sources may not be sufficient. Healthcare professionals may also need to help patients identify reliable resources, interpret health information, develop realistic self-management goals, and gain confidence in applying this information to their daily routines.

### 4.1. Limitations

This study has several limitations. First, the cross-sectional design precludes temporal or causal conclusions. Second, convenience sampling from a single university hospital limits generalizability. The sample was relatively young (mean age = 38.28 ± 14.79 years), predominantly male (62.3%), and comparatively highly educated (48.4% university/graduate). Findings may not generalize to older adults, women, individuals with lower education or limited digital access, or patients receiving care outside university-hospital settings. These considerations are particularly relevant because perceived eHealth literacy may vary with age, education, digital access, and prior technology exposure. Age at diagnosis was not available as a separate variable, limiting characterization of early-onset type 2 diabetes despite evidence that age at diagnosis is clinically relevant [21,22].

Third, eHEALS and DSES are self-report instruments completed during the same assessment period. Shared measurement method may contribute to their observed association. No formal common-method-variance assessment, including Harman’s single-factor test, was conducted; moreover, such a test would not definitively exclude common-method bias. Future studies should use temporal separation, multiple data sources, and performance-based measurements where possible. This issue is especially relevant when interpreting the eHEALS–DSES association because both constructs were assessed concurrently by self-report.

Fifth, the eHEALS measures perceived ability to find and use online health information rather than actual eHealth skills. Moreover, the scale does not provide separate scores for specific competencies such as critical appraisal and practical application. Consequently, the present study cannot determine whether participants had deficiencies in particular eHealth literacy domains. Norman [26] has noted the limitations of relying exclusively on perceived eHealth literacy, and the very high Cronbach’s alpha (0.951) may reflect some degree of item redundancy in addition to good reliability. Detailed data on device ownership, internet-use frequency, and digital-health engagement were not available for contextual analysis.

Fourth, exact numbers and patterns of discordant HbA1c/FPG classifications were not retained in a form that allowed reliable reconstruction for this revision; concordant and discordant participants could not be compared, and an HbA1c-only sensitivity analysis could not be performed. The robustness of the findings to alternative classification strategies therefore remains uncertain. Fifth, MCAR was not formally assessed, and listwise deletion may have introduced selection bias. Finally, important variables including treatment regimen, medication adherence, physical activity, dietary habits, socioeconomic circumstances, social support, and healthcare access were not assessed. In addition, the exact HbA1c-to-questionnaire interval and complete regression-diagnostic results were unavailable. Variables statistically adjusted for were age, gender, education, eHealth literacy, DSES, disease duration, and comorbidity status, whereas treatment type, adherence, diet, physical activity, and frequency of internet use were not measured and remain potential sources of residual confounding.

Longitudinal studies should measure eHealth literacy, self-efficacy, self-management behaviors, and glycemic outcomes at multiple time points. Such studies would help clarify whether eHealth literacy precedes changes in self-efficacy and whether these changes are subsequently associated with improvements in HbA1c. Randomized controlled trials are also needed to evaluate whether interventions combining digital health literacy education with self-efficacy-enhancing strategies improve diabetes self-management and glycemic outcomes compared with standard diabetes education.

Future research should use both perceived and performance-based measures of eHealth literacy and directly assess potential behavioral mechanisms, including medication adherence, dietary behavior, physical activity, glucose monitoring, and engagement with healthcare services. Studies involving multiple centers and participants from different age, educational, socioeconomic, and digital-experience groups would also strengthen the generalizability of the findings.

### 4.2. Conclusions

This study provides correlational evidence that eHealth literacy is associated with diabetes self-efficacy and that this relationship may partly account for the observed statistical association between eHealth literacy and HbA1c. Disease duration and self-efficacy were also associated with HbA1c. However, the cross-sectional design precludes conclusions regarding temporal or causal mediation, and modest R^2^ values limit the explanatory and predictive utility of the models.

The findings support consideration of digital health literacy and self-efficacy within diabetes education, but they do not establish that modifying either factor will improve glycemic control. Longitudinal and intervention studies are required to test these possibilities.

### 4.3. Implications and Recommendations for Practice

The observed associations suggest potential, but not proven, applications for diabetes care. Healthcare professionals could consider brief validated screening of perceived eHealth literacy; incorporate digital-literacy components into diabetes education; teach patients how to evaluate online diabetes information; demonstrate reliable digital resources; and tailor support according to age, education, access, and technology experience.

Simplified interfaces, guided practice, or additional support may be useful for individuals with lower perceived digital literacy. These approaches should complement, rather than replace, clinician–patient communication.

Self-efficacy-enhancing strategies such as goal setting, graded practice, problem-solving, self-monitoring, constructive feedback, and reinforcement may be combined with digital health literacy support. These are potential applications informed by observed associations, not interventions proven by this cross-sectional study to improve HbA1c.

Clinical approaches should consider age, education, digital access, socioeconomic resources, health status, and prior technology experience. The complexity, format, and delivery of digital education should be tailored to individual needs.

Longitudinal studies and randomized trials are required to determine whether strengthening eHealth literacy and self-efficacy improves self-management or glycemic outcomes.

## Figures and Tables

**Table 1:** Descriptive Characteristics of the Total Sample (*N* = 273).

Variable	*n* (%)/Valid *n*	Mean ± SD
Age (years)	273	38.28 ± 14.79
Gender—Male	170 (62.3)	
Gender—Female	103 (37.7)	
Education—Primary/Secondary	86 (31.5)	
Education—High School	55 (20.1)	
Education—University/Graduate	132 (48.4)	
Glycemic Status—Diabetes	163 (59.7)	
Glycemic Status—Prediabetes	110 (40.3)	
BMI (kg/m^2^)	268	27.09 ± 6.34
Waist Circumference (cm)	244	93.31 ± 25.63
HbA1c (%)	262	7.40 ± 1.86
FPG (mg/dL)	266	144.91 ± 57.18
Disease Duration (years)	252	5.86 ± 6.72
Comorbidity—Present	110 (40.3)	
eHEALS Total	273	25.77 ± 7.56
DSES Total	273	59.66 ± 12.93

Note: BMI = Body Mass Index; FPG = Fasting Plasma Glucose; eHEALS = eHealth Literacy Scale; DSES = Diabetes Self-Efficacy Scale.

**Table 2:** Mann–Whitney U and Chi-Square Comparisons Between Participants with Prediabetes and Type 2 Diabetes.

Variable	Prediabetes (*n* = 110)	Diabetes (*n* = 163)	U/χ^2^	*p*	|r|/V
Age (years)	41.75 ± 14.09	35.94 ± 14.84	11,060.0	0.001 **	0.23
BMI (kg/m^2^)	28.76 ± 6.28	25.92 ± 6.14	11,126.5	<0.001 ***	0.28
Waist Circ. (cm)	94.96 ± 17.64	92.01 ± 30.46	8546.5	0.026 *	0.17
HbA1c (%)	6.02 ± 0.24	8.38 ± 1.89	—	—	—
FPG (mg/dL)	108.50 ± 8.40	170.21 ± 62.71	—	—	—
Duration (years)	2.64 ± 4.01	8.28 ± 7.32	3688.0	<0.001 ***	0.53
eHEALS Total	25.42 ± 6.68	26.01 ± 8.11	7947.5	0.111	0.11
DSES Total	63.64 ± 11.66	56.97 ± 13.08	11,735.5	<0.001 ***	0.31
Gender (male %)	59.1	64.4	0.58	0.445	0.05

Note: U = Mann–Whitney U; |r| = rank-biserial correlation; χ^2^ = chi-square; V = Cramér’s V. HbA1c/FPG were used for classification; tests were not applicable. * *p* < 0.05, ** *p* < 0.01, *** *p* < 0.001.

**Table 3:** Spearman Rank-Order Correlations Among the Principal Continuous Study Variables.

Variable	1	2	3	4	5
1. Age	—				
2. eHEALS	−0.350 ***	—			
3. DSES	−0.052	0.310 ***	—		
4. HbA1c	−0.318 ***	0.176 **	−0.197 **	—	
5. FPG	−0.016	−0.053	−0.227 ***	0.552 ***	—

Note: Pairwise deletion was used because the number of available observations differed across variables (age, eHEALS, and DSES: *n* = 273; HbA1c: *n* = 262; FPG: *n* = 266). ** *p* < 0.01, *** *p* < 0.001.

**Table 4:** Multiple Linear Regression Analysis of Factors Associated With Diabetes Self-Efficacy in the Total Sample (*N* = 273).

Variable	B (SE)	β	*p*	t	VIF
Age	0.079 (0.059)	0.090	0.182	1.338	1.36
Gender	0.707 (1.548)	0.027	0.649	0.456	1.01
Education	−0.303 (1.063)	−0.021	0.776	−0.285	1.56
eHEALS	0.589 (0.115)	0.345 ***	<0.001	5.109	1.36

Note: Outcome = DSES total score. R^2^ = 0.102; F(4, 268) = 7.58, *p* < 0.001. B = unstandardized coefficient; SE = standard error; β = standardized coefficient. Gender: 1 = male, 2 = female. *** *p* < 0.001.

**Table 5:** Multiple Linear Regression Analysis of Factors Associated with HbA1c Among Participants With Type 2 Diabetes (*n* = 134).

Variable	B (SE)	β	*p*	t	VIF
Age	−0.019 (0.016)	−0.154	0.232	−1.201	2.44
Gender	0.302 (0.326)	0.079	0.356	0.926	1.08
Education	0.393 (0.279)	0.192	0.162	1.406	2.77
eHEALS	0.019 (0.027)	0.088	0.474	0.718	2.23
DSES	−0.031 (0.014)	−0.224 *	0.026	−2.252	1.47
Duration	0.053 (0.023)	0.201 *	0.023	2.295	1.14
Comorbidity	0.143 (0.387)	0.039	0.713	0.368	1.65

Note: Outcome = HbA1c. Expanded model R^2^ = 0.155; F(7, 126) = 3.29, *p* = 0.003. The basic model without disease duration and comorbidity had R^2^ = 0.103. B = unstandardized coefficient; SE = standard error; β = standardized coefficient. Gender: 1 = male, 2 = female. * *p* < 0.05.

**Table 6:** Bootstrapped Statistical Indirect-Association Analysis Between eHealth Literacy and HbA1c Through Diabetes Self-Efficacy Among Participants With Type 2 Diabetes.

Path	Coefficient	SE	*p*	95% Boot CI
a (eHEALS → DSES)	0.644	0.115	<0.001 ***	—
b (DSES → HbA1c)	−0.034	0.009	<0.001 ***	—
c (total effect)	0.002	—	—	—
c′ (direct effect)	0.024	—	ns	—
Indirect (a × b)	−0.022	—	0.002 **	[−0.039, −0.009]

Note: Bootstrap resamples = 5000. Analyses controlled for age and education. ** *p* < 0.01, *** *p* < 0.001.

## Data Availability

The data presented in this study are available from the corresponding author upon reasonable request. The data are not publicly available because they contain participant-level clinical information and are subject to the conditions of the ethics approval and informed consent.
